# Electron Beam Induced Artifacts During *in situ* TEM Deformation of Nanostructured Metals

**DOI:** 10.1038/srep16345

**Published:** 2015-11-10

**Authors:** Rohit Sarkar, Christian Rentenberger, Jagannathan Rajagopalan

**Affiliations:** 1 School for Engineering of Matter Transport and Energy, Arizona State University, Tempe 85287, USA; 2 Physics of Nanostructured Materials, Faculty of Physics, University of Vienna, Boltzmanngasse 5, 1090 Vienna, Austria

## Abstract

A critical assumption underlying *in situ* transmission electron microscopy studies is that the electron beam (e-beam) exposure does not fundamentally alter the intrinsic deformation behavior of the materials being probed. Here, we show that e-beam exposure causes increased dislocation activation and marked stress relaxation in aluminum and gold films spanning a range of thicknesses (80–400 nanometers) and grain sizes (50–220 nanometers). Furthermore, the e-beam induces anomalous sample necking, which unusually depends more on the e-beam diameter than intensity. Notably, the stress relaxation in both aluminum and gold occurs at beam energies well below their damage thresholds. More remarkably, the stress relaxation and/or sample necking is significantly more pronounced at lower accelerating voltages (120 kV versus 200 kV) in both the metals. These observations in aluminum and gold, two metals with highly dissimilar atomic weights and properties, indicate that e-beam exposure can cause anomalous behavior in a broad spectrum of nanostructured materials, and simultaneously suggest a strategy to minimize such artifacts.

Metallic materials with sub micrometer microstructural or sample dimensions exhibit mechanical properties that are distinct from their bulk, coarse-grained counterparts^1^,^2^,^3^,^4^,^5^,^6^,^7^,^8^,^9^,^10^. Transmission electron microscopy (TEM), because of its atomic scale resolution and the capability to measure crystal rotations and lattice strains using diffraction, has been extensively used to understand the deformation behavior of such materials^11^,^12^,^13^,^14^,^15^,^16^,^17^. In particular, *in situ* TEM straining experiments have provided insights into the deformation mechanisms^18^,^19^,^20^, inelastic strain recovery^21^,^22^, grain rotation and growth^23^,^24^,^25^, and generation and motion of dislocations^26^,^27^,^28^ in nanocrystalline and ultrafine-grained metals. More recently, a combination of conventional TEM and automated crystal orientation mapping has enabled further insights into the mechanisms of grain growth and deformation^29^,^30^,^31^.

While TEM is a fundamental technique to characterize nanoscale materials, it is also well known that the high energy of the electron beam (e-beam) used in TEM can cause radiation damage in the materials being probed^32^. In addition, the e-beam can activate dislocations, and defects generated by e-beam exposure can lead to additional deformation processes. For example, low to moderate intensity e-beam has been used to induce superplastic deformation in nanoscale silica particles and nanowires that are usually brittle at low temperatures^33^. Since nanocrystalline and ultrafine-grained metals have a high density of crystal defects (non-equilibrium grain boundaries and associated extrinsic dislocations) that can be activated by the e-beam, it is quite conceivable that e-beam exposure will alter the mechanical behavior of such materials during *in situ* experiments. However, the e-beam effect on the *in situ* deformation response of nanostructured materials remains unknown.

Here, using quantitative *in situ* TEM tensile straining of aluminum and gold films with a range of thicknesses (80–400 nm) and mean grain sizes (*d* ~ 50–220 nm) we provide direct evidence that e-beam exposure causes increased dislocation activation, significant stress relaxation and anomalous changes in sample geometry. By systematically controlling the beam conditions (accelerating voltage, intensity and beam diameter) during cyclic deformation and stress relaxation experiments we show that, contrary to expectation, beam-induced artifacts are much more pronounced at lower accelerating voltages (120 kV compared to 200 kV). In addition, the experiments reveal that e-beam exposure causes an unexpected necking of the samples along their width, which depends more on the beam diameter than intensity. These observations in two metals with highly dissimilar atomic weights and properties strongly suggest that the e-beam can significantly alter the deformation response of a broad spectrum of nanostructured materials. At the same time, the results also point to strategies that can be adopted to minimize beam-induced artifacts during *in situ* TEM testing.

## Experimental

**Figure 1:**
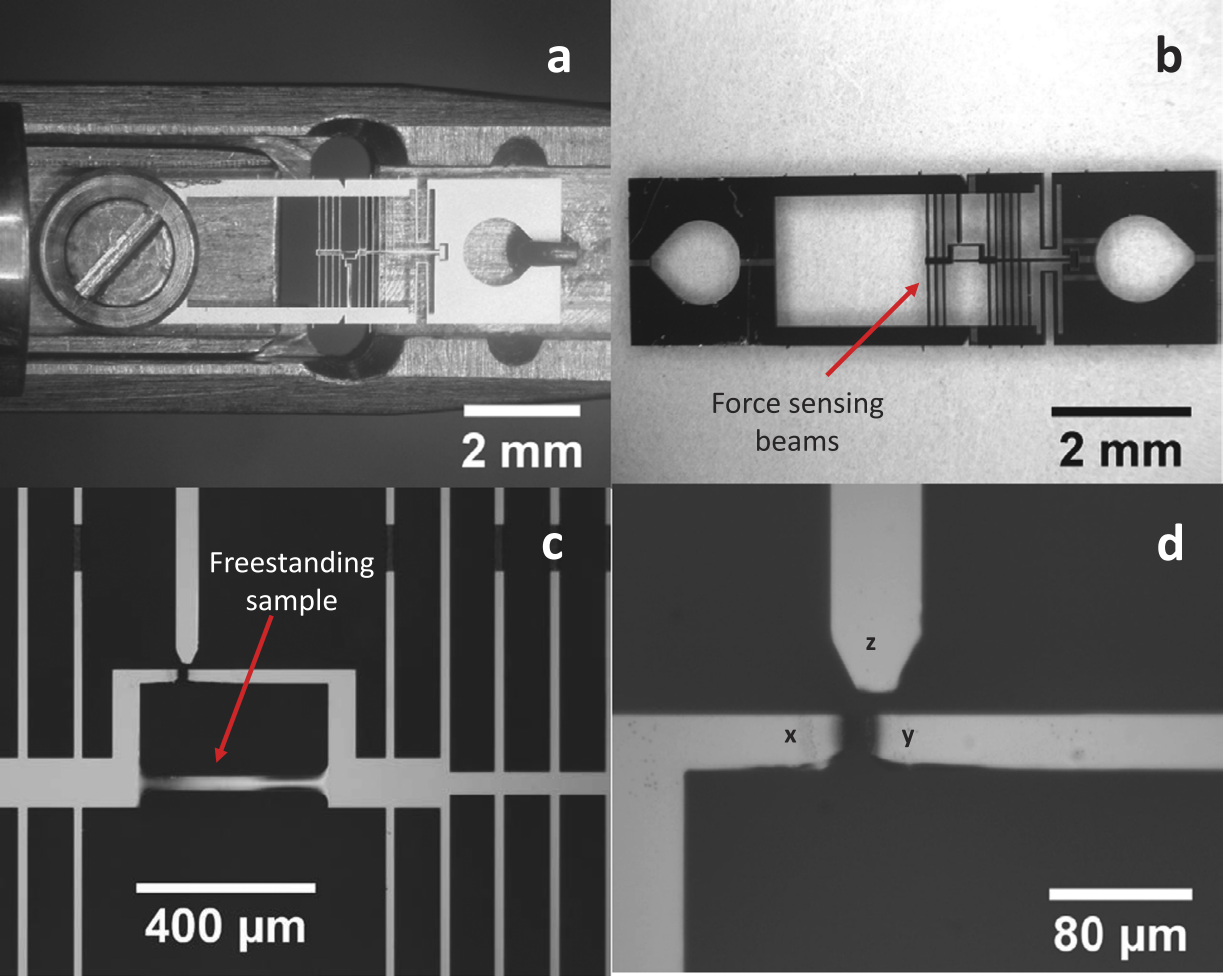
MEMS device and experimental setup: (**a**) Optical micrograph of a typical MEMS device mounted on the TEM straining holder. (**b**) Optical micrograph of the MEMS device showing the force sensing beams. (**c**) Optical micrograph showing the freestanding Al sample and the strain and force measuring gauges. (**d**) Zoomed in image of the two strain sensing gauges *x* and *y* and the stationary force sensing gauge, *z*. The change in distance between gauges *x* and *y* gives the deformation on the sample while the change in distance between gauges *x* and *z* multiplied by the stiffness of the force sensing beams gives the force acting on the sample.

Two Al films, 225 nm and 400 nm thick, respectively, and one Au film, 80 nm thick, were deposited on 200-μm thick, 100 mm diameter, (100) silicon (Si) wafers using DC magnetron sputtering. Before deposition, the Si wafers were etched with hydrofluoric acid to remove the native silicon dioxide layer. The 225 nm thick Al film (from here on referred to as Al-225 film) and Au film were deposited immediately after the removal of the silicon dioxide layer at base pressures of 1.5 × 10^−8^ Torr and 5 × 10^−8^ Torr respectively. The 400 nm thick Al film (from here on referred to as Al-400 film) film was deposited at a base pressure of 1.5 × 10^−7^ Torr about 1.5 hours after the removal of the oxide layer. For all depositions, the argon pressure was 3 mTorr and the deposition rate was between 4–5 nm/minute. Dog-bone shaped freestanding samples were then co-fabricated with micro-electro-mechanical systems (MEMS) based tensile testing devices (Fig. 1) using photolithography and plasma etching techniques described in^34^. The MEMS devices, with a width of 2.5 mm and length of 9 mm, have built-in force and displacement sensing gauges to measure the stress and strain during *in situ* TEM straining. The effective gauge length of all the samples was 395 μm, whereas the width was 30 μm.

The MEMS devices with the freestanding film samples were mounted on a displacement controlled single tilt straining holder and the films were subjected to cyclic deformation inside a Philips CM200 TEM equipped with a LaB_6_ cathode. In each of the straining cycles the beam condition was systematically controlled as follows. The beam intensity was varied for a given beam area by changing the e-beam current. The beam current was controlled by altering the Wehnelt bias, the filament heating current of the TEM and the size of the condenser aperture. In all the experiments the microstructure was observed under TEM bright field conditions and recorded using an Orius Gatan^TM^ CCD camera.

Strain pulses (typically corresponding to <0.1% strain) were applied to the sample, following which the sample was allowed to relax for a period of 5 minutes and the stress-strain data was then recorded. Two types of experiments were performed to quantify the effect of e-beam exposure. In the first set of experiments, the stress-strain response of the Al-225, Al-400 and Au film during the first cycle was measured without e-beam exposure to serve as a reference. In the subsequent cycles, the films were exposed to the e-beam at different accelerating voltages and the beam diameter (area) and intensity were systematically varied. In the second set of experiments on the Al-225 film, the sample was exposed to the e-beam only at two points during each loading to quantify beam-induced stress relaxation. At both these points, stress-strain data was recorded for a period of 15 minutes, typically at 5-minute intervals. Table 1 and 2 show the beam conditions for all the experiments.

The stress-strain response of the films was obtained by analyzing images of the built-in force and displacement gauges during deformation, using a custom MATLAB^TM^ program. The program tracks prescribed features using cross-correlation techniques across a series of images to calculate the displacement of the gauges. Using this process, stress and strain resolutions better than 2 MPa and 0.005%, respectively, were obtained for both Al and Au films.

Once the raw stress-strain data was obtained, the data was fitted with polynomial functions to obtain the stress at a given strain. To avoid over fitting, for both loading and unloading the polynomial order was chosen so that there were at least three data points for each coefficient. Typically, fourth order polynomials were used to fit the stress-strain data for loading and third order polynomials were used for unloading. For stress-relaxation, for which only four or fewer data points were measured, a linear fit was chosen. The R^2^ value was higher than 0.995 for all the loading and unloading fits. Similarly, the R^2^ value for fits to the stress relaxation data exceeded 0.98 except for the first stress drop in the 2^nd^ cycle (R^2^ = 0.804), when the relaxation was very small (Table 2). The equations of the fitted curves were then used to extract the stress at 1% strain (σ_1%_) for the Al-225 and Au films, the stress at 0.75% strain (σ_0.75%_) for the Al-400 film and the stress drop for relaxation experiments on the Al-225 film. σ_1%_ and σ_0.75%_ were calculated for a particular cycle after offsetting the stress-strain curve to account for residual plastic strain from previous cycles. To ensure robustness of the analysis we also calculated σ_1%_ and σ_0.75%_ from higher order and lower order polynomial fits, but observed no meaningful changes.

## Results

**Figure 2:**
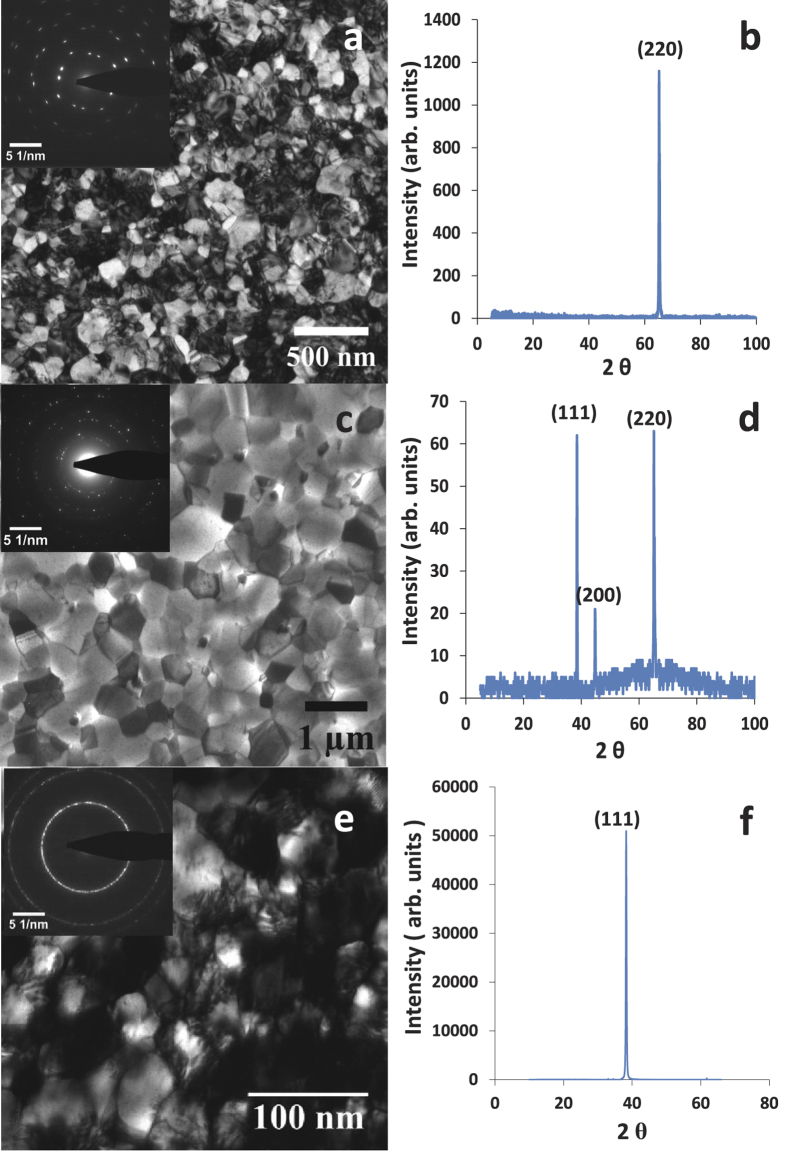
Microstructure of Al and Au films: (**a**) Bright-field TEM image and selected area diffraction (SAD) pattern (inset) of the undeformed Al-225 film (*d* = 120 nm). The SAD pattern shows an (110) out-of-plane texture with two in-plane grain variants rotated 90^0^ with respect to each other. (**b**) XRD scan of the Al-225 film. (**c**) Bright-field TEM image of the undeformed Al-400 film (*d* = 220 nm). The ring like SAD pattern (inset) reveals random in-plane grain orientations. (**d**) XRD scan of the Al-400 film. The (111) and (220) peaks have similar intensities, whereas in random polycrystalline Al their ratio is 10:3, which shows a mild (110) out-of-plane texture. (**e**) Bright-field TEM image and SAD pattern (inset) of the undeformed Au film (*d* = 50 nm). (**f**) XRD scan of the Au film showing an (111) out-of-plane texture.

The microstructure of the films was characterized by TEM and x-ray diffraction (XRD). The analyses (Fig. 2a,b) revealed a *d* of 120 nm and an (110) out-of-plane texture with only two in-plane grain variants (bicrystalline microstructure) for the Al-225 film. The Al-400 film had a higher *d* of 220 nm and a mild (110) out-of-plane texture with random in-plane grain orientations (Fig. 2c,d), whereas the Au film had a *d* of 50 nm and a strong (111) out-of-plane texture with random in-plane grain orientations (Fig. 2e,f).

**Figure 3:**
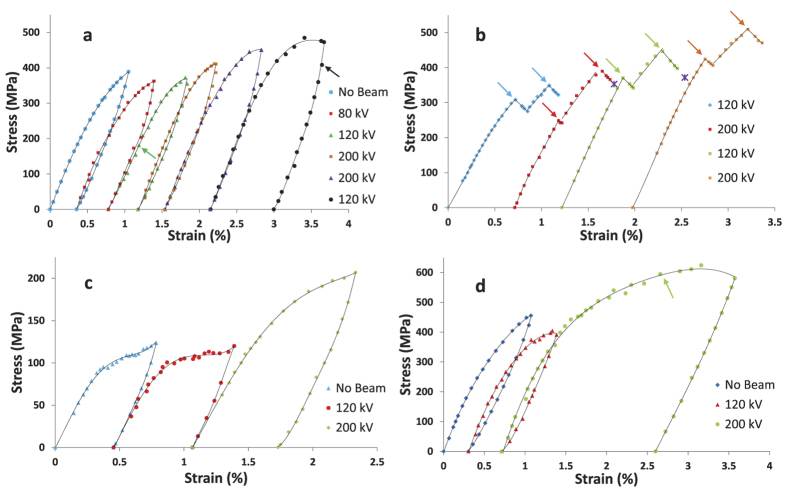
Cyclic stress-strain response and stress relaxation of Al and Au films: (**a**) Stress-strain response of the Al-225 film over six cycles under different beam conditions. A reduction in stress can be observed for the 2^nd^ and 3^rd^ cycle with respect to the 1^st^ cycle. The imaging conditions for the different deformation cycles are summarized in Table 1. The green and black arrows correspond to Fig. 4 and supplementary video 2 (videoS2), respectively. (**b**) Beam induced stress relaxation in the Al-225 film. The film was exposed to the e-beam only at two points during each loading (indicated by arrows). At the end of the 2^nd^ and 3^rd^ cycles, the e-beam was moved to a new location, which led to significant additional relaxation (indicated by the violet cross). (**c**) Stress-strain response of the Al-400 film over three cycles with different beam conditions. (**d**) Stress-strain response of the Au film over three cycles with different beam conditions. The green arrow in (**d**) corresponds to supplementary video 4 (videoS4). In all the figures, the black lines correspond to fitted polynomials while the individual points indicate raw data.

Figure 3 shows the stress-strain response of the films for the cyclic loading and stress relaxation experiments under different beam conditions. To quantify the e-beam effect on the stress-strain response during the cyclic loading experiments, we compare the stress at the same strain level during each cycle, after offsetting the stress-strain curve to account for residual plastic strain from previous cycles. For the Al-225 and Au film, σ_1%_ is used for comparison. The overall trend is very similar even if we use the stress at a lower strain level for comparison. The Al-400 film started deforming plastically at lower strain and hence was subjected to smaller strains during each cycle. Therefore, we compare the σ_0.75%_ during each cycle. σ_1%_ for the Al-225 and Au film and σ_0.75%_ for the Al-400 film during each cycle is summarized in Table 1.

In cyclic loading of metal films, the stress for a given strain is usually higher in the later cycles due to strain hardening. However, σ_1%_ for the Al-225 film (Table 1, Fig. 3a) decreased from 380 MPa in the 1^st^ cycle (no e-beam exposure) to 358 MPa in the 2^nd^ cycle (80 kV beam). Notably, σ_1%_ for the 3^rd^ cycle (120 kV beam) was still ~5% lower than the 1^st^ cycle despite the significant plastic deformation induced in the film during the first two cycles. This clearly shows the effect of e-beam exposure on the stress-strain response of the Al-225 film. When the accelerating voltage was increased to 200 kV in the 4^th^ cycle, σ_1%_ was markedly higher (408 MPa) compared to both the 3^rd^ and 1^st^ cycle. Between the 4^th^ and 5^th^ cycle, the accelerating voltage and beam current (area times intensity) were kept constant but intensity was reduced 4-fold, which resulted in a slight increase of σ_1%_ to 419 MPa. In the 6^th^ cycle, the accelerating voltage (120 kV) and beam current were made identical to the 3^rd^ cycle but the intensity was reduced 4-fold by spreading the beam. In addition, the e-beam was shifted to a previously unexposed region of the sample. This led to an increase in σ_1%_ to 445 MPa.

The marked increase in σ_1%_ of the Al-225 film between the 3^rd^ and 4^th^ cycle (Table 1) suggested that beam-induced stress relaxation is higher at 120 kV compared to 200 kV. However, the e-beam intensity and current were different for those cycles and hence a definitive conclusion could not be made. To unambiguously verify if stress relaxation is higher at 120 kV, we performed stress relaxation experiments on a different sample of the Al-225 film (Fig. 3b) at 120 kV and 200 kV. In the first two cycles, we kept the e-beam area, intensity and current identical and just varied the accelerating voltage. We loaded the sample without e-beam exposure and irradiated the sample with the e-beam at only two points during loading and measured the percentage stress drop (decrease in stress/initial stress) on each occasion. As evident from the data (Fig. 3b, Table 2), stress relaxation was significantly higher at 120 kV. In the 3^rd^ and 4^th^ cycle we increased the beam area and decreased the intensity while keeping the beam current close to its value in the 1^st^ and 2^nd^ cycle. Again, the stress relaxation was higher at 120 kV (3^rd^ cycle), even though the intensity was slightly larger in the 4^th^ cycle (200 kV).

To ensure that the larger stress relaxation observed in the Al-225 film at lower voltages is not unique to a particular grain size, sample texture or thickness, we repeated the cyclic load-unload experiments on a thicker Al film (Al-400). This film had a notably larger mean grain size (*d* = 220 nm) compared to the Al-225 film (*d* = 120 nm) and the texture was also different (Fig. 2). Despite these differences, the Al-400 film also showed a significantly larger stress relaxation at 120 kV (Fig. 3c, Table 1) compared to 200 kV. In fact, the percentage decrease in σ_0.75%_ for the Al-400 film at 120 kV was even higher compared to the percentage decrease in σ_1%_ for the Al-225 film at the same voltage (3^rd^ cycle in Fig. 3a).

We further verified if this trend (higher stress relaxation at lower voltages) was consistent across different materials by performing cyclic load-unload experiments on an Au film. The Au film had a considerably different texture and a much smaller mean grain size (Fig. 2) and thickness compared to both the Al-225 and Al-400 film. Nevertheless, the e-beam effect on the stress-strain response of the Au film (Fig. 3d) was fully consistent with the effect on the Al films, leading to significantly higher stress relaxation at 120 kV compared to 200 kV.

**Figure 4:**
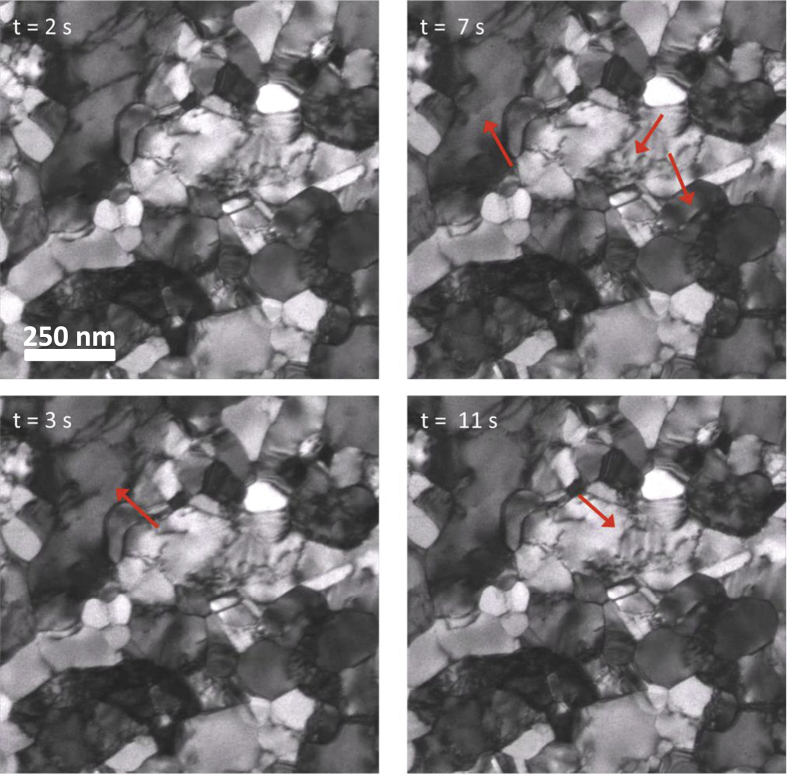
Dislocation motion during straining: TEM bright-field images of the Al-225 film after the application of a set of displacement pulses during the 3^rd^ cycle. The approximate stress and strain at this point was 225 MPa and 0.41%, respectively (green arrow in Fig. 3a). The red arrows point to locations of dislocation activity. The video corresponding to these images (videoS1) is available as supplementary material.

In addition to the stress-strain response, the evolution of the microstructure during and after deformation was also monitored. The Al-225 film showed a small increase in *d* from 120 nm to 135 nm after six cycles whereas the Al-400 and Au film did not show any grain growth. In all the films dislocation activity was seen even at small strains where the stress-strain curve showed little deviation from linearity. Figure 4 shows an example of such dislocation activity in the Al-225 film during the third deformation cycle when the strain was ~0.41% (indicated by the green arrow in Fig. 3a). The video corresponding to these images (videoS1) is available as supplementary material. Notably, significant dislocation activity was also seen during the initial stages of unloading (videoS2), when the macroscopic stress-strain response was elastic. The stress corresponding to videoS2 is about 408 MPa (indicated by the black arrow in Fig. 3a), 76 MPa lower than the peak stress during the cycle. These observations suggest that dislocations are activated by e-beam exposure, leading to localized plastic deformation.

To confirm this, during the stress relaxation experiments on the Al-225 film (Fig. 3b) we shifted the e-beam to a different region of the sample at the end of 2^nd^ loading cycle for approximately 2 minutes. Immediately upon e-beam exposure, substantial dislocation activity (videoS3) was observed in the new region, which was accompanied by further stress relaxation (violet cross at the end of 2^nd^ cycle). Note that this additional stress relaxation occurred even though the sample had been exposed to the e-beam for 15 minutes in the previous observational area and significant stress relaxation (>6%) had already occurred. We similarly shifted the beam to a new location at the end of the 3^rd^ cycle and again observed significant additional relaxation (violet cross at the end of 3^rd^ cycle). To verify if the same e-beam induced mechanism is active in the Au film, we temporarily shifted the beam to a new location during the 3^rd^ loading cycle (indicated by green arrow in Fig. 3d). Once again, this led to enhanced dislocation activation in the new location (videoS4).

**Figure 5:**
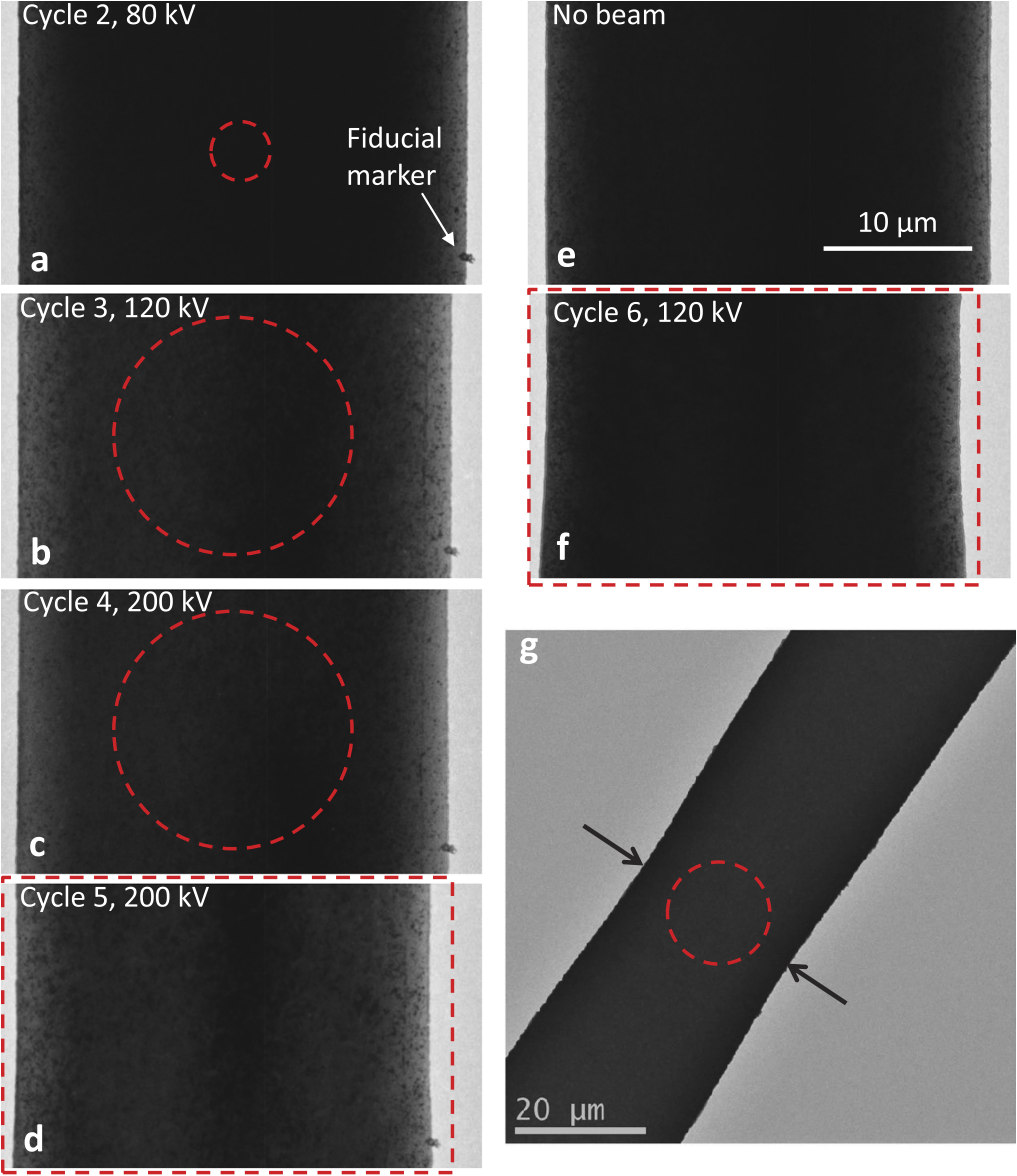
Anomalous necking of films: (**a**–**f**) TEM images of the Al-225 sample at the locations where it was exposed to the e-beam, with scale bar shown in (**e**). The left edge of the sample is aligned so that changes in width after each cycle can be easily seen. The red circles in (**a**–**c**) are the areas exposed to the e-beam. In (**d**) and (**f**), the entire width of the sample was exposed as indicated by the red rectangles. The center of the e-beam was approximately at the same location from the 2^nd^ to 5^th^ cycle. The imaging location was changed before the 6^th^ cycle. The image in (**e**) corresponds to a previously unexposed region of the sample before the 6^th^ cycle. The image in (**f**) corresponds to the region in (**e**) after exposure to the e-beam during the 6^th^ cycle. The necking of the sample is clearly visible in (**d**) and (**f**). (**g**) TEM image of the 400 nm sample after the 2^nd^ cycle, with the arrows pointing to the necked region.

Apart from microstructural changes, significant necking along the sample width was seen in the Al-225 film (Fig. 5). This localized necking exactly corresponded to the location of the e-beam on the sample (indicated by a red circle or rectangle), which strongly suggests that the necking is a direct consequence of e-beam exposure. In the Al-225 film, the width reduced from 30 μm to 27.54 μm, an 8.2% reduction, after 5 deformation cycles. To understand the effect of beam conditions on necking, we analyzed the changes in sample width (*Δw*) from the 2^nd^ to 5^th^ cycle. Since the initial sample width (at the beam location) was different for each cycle, we first calculated the percentage width reduction (*Δw*/*w*) for each cycle. We then normalized *Δw*/*w* by the plastic strain (*ε*_*p*_) imposed in that cycle. After this normalization, we found a clear dependence of necking on both the beam area as well as accelerating voltage (Table 3).

When the beam area was very small (2^nd^ cycle) there was no notable decrease in the sample width. For a given accelerating voltage and beam current (4^th^ and 5^th^ cycle), the normalized width reduction (*r* = (*Δw*/*w*)/*ε*_*p*_) was significantly larger when the beam area was larger. This suggests that the extent of necking is more dependent on the beam area than intensity. Similar to stress relaxation, necking was also more pronounced at 120 kV. For example, in the 3^rd^ cycle (120 kV beam) *r* = 8.35, which is higher by a factor of 2.5 compared to the 4^th^ cycle (200 kV beam, *r* = 3.28), even though the beam area was the same and beam intensity was only 1.7 times larger. Interestingly, when the e-beam was shifted to a new region for the 6^th^ cycle (120 kV), a new neck appeared in that region and there was no further reduction in width of the previously necked region, which confirms that necking results from e-beam exposure. Furthermore, the specimen width decreased from 30 μm to 27.54 μm (8.2% reduction) in a single cycle.

Similar sample necking was seen in the Al-400 film as well (Fig. 5g) but the reduction in width was even more pronounced. The sample width reduced from 30 μm to 26.85 μm (10.5% reduction) after just one cycle of deformation at 120 kV, even though the imposed plastic strain was only 0.6% and the beam intensity was also quite low (Table 1). In the Au film no obvious necking was observed, presumably because the beam area was much smaller (Table 1).

## Discussion

Radiolysis, caused by the inelastic scattering of incident beam electrons, and knock-on displacement, due to elastic scattering of beam electrons, are the two main radiation damage mechanisms in TEM. However, in conducting materials such as metals, radiolysis is mostly suppressed because of the high density of delocalized electrons^32^, which leaves knock-on displacement as the primary damage mechanism. Since knock-on displacement typically increases with accelerating voltage, a maximum voltage of 200 kV is used in most *in situ* TEM deformation experiments on metals to mitigate radiation damage. Nevertheless, recent studies have shown that even low to moderate e-beam exposure in a TEM can affect the mechanical behavior of nanoscale materials such as nanoparticles and nanowires^33^,^35^.

Our experiments demonstrate that the mechanical behavior of nanocrystalline and ultrafine-grained metals is also significantly affected by electron irradiation. In particular, the experiments reveal two unexpected trends as discussed below. First, beam-induced artifacts in nanostructured metals occur at beam energies far below the radiation damage (knock-on displacement) thresholds of these materials. Second, the beam-induced artifacts are more pronounced at lower accelerating voltages.

Knock-on displacement occurs when the beam energy exceeds a threshold, and may either result in the formation of vacancy-interstitial pairs in the bulk or lead to sputtering at the surfaces. However, the threshold energy for knock-on displacement in the bulk is usually larger than at the surface. For Al, the bulk displacement threshold energy exceeds 100 kV^36^ but the surface sputtering threshold energy is only 65 kV^37^. Since substantial stress relaxation is observed at 80 kV for the Al-225 sample (Fig. 3a), it is conceivable that surface sputtering could be partly responsible for the observed relaxation. However, the experiments on the Al-400 film (Fig. 3c) strongly suggest otherwise. If surface sputtering were a primary damage mechanism, the e-beam effect would be much smaller on the Al-400 film because of its higher thickness. In contrast, stress relaxation as well as necking in the Al-400 film (at 120 kV) was more pronounced compared to the Al-225 film.

The experiments on the Au film provide even stronger evidence that knock-on displacement is not responsible for the observed stress relaxation. The estimated surface sputtering threshold energy for Au is around 400 kV^37^ and the threshold for knock-on displacement in the bulk exceeds 1000 kV. Therefore, no e-beam effect would be expected at 120 kV. Still, considerable stress relaxation (>10%) was observed when the film was exposed to the 120 kV beam (Fig. 3d and Table 1).

The experiments also reveal that the e-beam effects are more pronounced at 120 kV compared to 200 kV for all three films and the results are consistent across both cyclic loading as well as stress relaxation experiments (Fig. 2, Table 1). For example, in the Al-400 film σ_0.75%_ decreased by 9% in the 2^nd^ cycle compared to the 1^st^ cycle (no beam) due to irradiation by 120 keV electrons, whereas σ_0.75%_ increased by ~40% when 200 keV electrons were used to image the sample in the 3^rd^ cycle. Thus, there was a qualitative change in the stress-strain response when the accelerating voltage was changed, even though the beam areas were identical and beam intensities were comparable. The stress-strain behavior of the Au film (Fig. 3d, Table 1) provides even more convincing evidence that the e-beam effect is higher at lower voltages since in this case the beam conditions (area, intensity and current) were identical. While σ_1%_ decreased by 11% between the 1^st^ cycle (no beam) and 2^nd^ cycle (120 kV beam), it was 5% higher in the 3^rd^ cycle (200 kV beam) compared to the 1^st^ cycle.

The trends in stress relaxation strongly indicate that knock-on displacement cannot explain the observations, which suggests that other processes are responsible for the changes in stress-strain behavior and sample geometry. It is well known that conduction electrons interact with lattice defects such as grain boundaries and dislocations and this leads to an increase in electrical resistivity^38^ compared to a perfect crystal. Furthermore, it has been shown that an electric current pulse of high density (10^4^ A/cm^2^) can assist dislocations in overcoming obstacles causing electroplasticity^39^.

In the present case, the incident beam electrons play a role analogous to the electric current pulse. The beam electrons scatter inelastically near lattice defects and generate local lattice vibrations (phonons) either by scattering-induced phonon generation or via the excitation and damping of plasmons^40^,^41^. The interaction of the phonons and dislocations leads to depinning of dislocations pinned at the grain boundaries. This phonon-assisted depinning increases dislocation mobility and consequently results in stress relaxation. For a given beam area, the number of inelastic scattering events increases with increasing beam intensity. Therefore, stress relaxation caused by phonon assisted depinning of dislocations increases with increasing beam intensity. Similarly, if the intensity is constant and the beam area increases, stress relaxation is again higher because dislocation activation is enhanced in a larger fraction of grains across the sample width.

Notably, this mechanism is consistent with the observation that when the beam is shifted to a new location there is increased dislocation activity in both the Al-225 film (videoS3) and the Au film (videoS4). In this context, it is also worth noting that the cross-section of inelastic processes typically decreases with increasing beam energy^42^. Therefore, the probability for phonon generation and dislocation activation would be higher at 120 kV compared to 200 kV, which can explain the higher e-beam effect at 120 kV. However, it is important to note that these phonons generated by inelastic scattering of beam electrons do not lead to a sustained increase in the overall sample temperature. Both Al and Au have high thermal conductivity (>200 W/m/K) and even the largest beam currents used in our experiments are not expected to raise the sample temperature by more than 1 K^42^. This remains the case even when we account for the lower thermal conductivity of nanocrystalline materials^43^.

In addition to stress relaxation, the e-beam also caused considerable necking of the films (Fig. 5). This necking along the width is highly unusual because in thin film samples with a relatively large width, the thickness tends to decrease more during uniaxial loading due to geometrical constraints (Supplementary Fig. 1). Similar to stress relaxation, the necking was also more pronounced at a lower accelerating voltage. But somewhat surprisingly the extent of necking was more sensitive to the beam area than beam intensity (compare 4^th^ and 5^th^ cycles or 3^rd^ and 6^th^ cycles of the Al-225 film). One possible explanation is that when the beam area is large, dislocation activation is increased in a higher fraction of grains across the sample width and these grains collectively deform to form a neck. When the beam area is small, plastic relaxation is enhanced in only a small fraction of grains across the sample. So even if the extent of relaxation in these grains is higher (because of higher beam intensity), their deformation is constrained by the surrounding grains that undergo no relaxation. Thus, there is less macroscopic necking of the sample.

Overall, these results unambiguously show that the beam energy required to induce stress relaxation in nanostructured metals is substantially lower than their radiation damage threshold and that the relaxation is more pronounced at lower beam energies. It is important to note that these beam-induced artifacts are observed in two metals (Al and Au) that have highly dissimilar atomic weights and substantial differences in material properties (stacking fault energy, for example). The observations are also consistent across a range of sample thicknesses and grain sizes. This strongly suggests that e-beam exposure is likely to alter the deformation behavior in a broad spectrum of nanostructured metals, including commonly studied metals such as nickel and copper.

Furthermore, our results show that e-beam exposure causes anomalous changes in geometry when a larger cross-sectional area of the sample is exposed. Therefore, materials with sub micrometer dimensions (nanowires, nano pillars), which typically have their entire cross-section illuminated by the e-beam during *in situ* deformation experiments, are likely to be more susceptible to e-beam induced artifacts. Hence, caution needs to be exercised in interpreting the stress-strain response of such materials from *in situ* experiments.

While the observations clearly demonstrate the e-beam effect on the deformation response of nanocrystalline and ultrafine-grained metals, they also suggest a strategy to minimize beam-induced artifacts during *in situ* testing. The results indicate that beam-assisted dislocation activation is far more important in these materials compared to radiolysis or knock-on damage. And since dislocation activation is reduced at higher beam energies, it may be prudent to employ higher accelerating voltages during *in situ* testing of nanostructured materials. However, it should be noted that the e-beam intensities used in this study are quite low (<1.5 A/cm^2^). When higher intensities (for high resolution imaging or spectroscopy applications) are employed, knock-on damage could become significant at higher accelerating voltages. Therefore, careful studies are required to establish appropriate imaging conditions to minimize beam-induced artifacts during *in situ* TEM deformation of nanocrystalline and ultrafine-grained metals.

## Additional Information

**How to cite this article**: Sarkar, R. *et al.* Electron Beam Induced Artifacts During *in situ* TEM Deformation of Nanostructured Metals. *Sci. Rep.* **5**, 16345; doi: 10.1038/srep16345 (2015).

## Supplementary Material

Supplementary Figure

Supplementary Video Captions

Supplementary Video 1

Supplementary Video 2

Supplementary Video 3

Supplementary Video 4

## Figures and Tables

**Table 1:** Beam conditions and stress response of Al-225, Al-400 and Au film during cyclic deformation.

Al-225	Cycle	Voltage (kV)	Area (μm^2^)	Current (nA)	Intensity (A/cm^2^)	σ_1%_ (MPa)
	1	0	0	0	0	380
	2	80	12.5	44	0.352	358
	3	120	201	103	0.051	362
	4	200	201	61	0.03	408
	5	200	804	61	0.007	419
	6	120	804	103	0.012	445
Al-400	Cycle	Voltage (kV)	Area (μm^2^)	Current (nA)	Intensity (A/cm^2^)	σ_0.75%_ (MPa)
	1	0	0	0	0	120
	2	120	201	14	0.0069	109
	3	200	201	10	0.0049	167
Au	Cycle	Voltage (kV)	Area (μm^2^)	Current (nA)	Intensity (A/cm^2^)	σ_1%_ (MPa)
	1	0	0	0	0	443
	2	120	7	95	1.357	395
	3	200	7	95	1.357	464

**Table 2:** Beam conditions and stress response of Al-225 film during relaxation experiments.

Cycle	Voltage (kV)	Area (μm^2^)	Current (nA)	Intensity (A/cm^2^)	1^st^ Stress Drop (%)	2^nd^ Stress Drop (%)
1	120	201	71	0.035	8.06	7.79
2	200	201	71	0.035	2.88	6.24
3	120	804	61	0.0075	7.37	10.63
4	200	804	66	0.0082	4.32	7.79

**Table 3:** Beam conditions and necking parameters of Al-225 film during cyclic deformation.

Cycle	Voltage (kV)	Area (μm^2^)	Intensity (A/cm^2^)	*ε*_*p*_ (%)	*w* (μm)	*Δw*(μm)	*Δw/w*(%)	r = 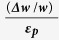
2	80	12.5	0.352	0.426	30	0.00	0.00	0.00
3	120	201	0.051	0.399	30	1	3.33	8.35
4	200	201	0.03	0.357	29	0.34	1.17	3.28
5	200	804	0.007	0.606	28.66	1.12	3.91	6.45
6	120	804	0.012	0.848	30	2.46	8.20	9.67

## References

[b1] Kumar K., Van Swygenhoven H. & Suresh S. Mechanical behavior of nanocrystalline metals and alloys Acta Mater. 51, 5743–5774 (2003).

[b2] Uchic M. D., Shade P. A. & Dimiduk D. M. Plasticity of Micrometer-Scale Single Crystals in Compression. Annu. Rev. Mater. Res. 39, 361–386 (2009).

[b3] Greer J. R. & De Hosson J. T. M. Plasticity in small-sized metallic systems: Intrinsic versus extrinsic size effect. Prog. Mater. Sci. 56, 654–724 (2011).

[b4] Schwaiger R., Moser B., Dao M., Chollacoop N. & Suresh S. Some critical experiments on the strain-rate sensitivity of nanocrystalline nickel. Acta Mater. 51, 5159–5172 (2003).

[b5] Wang Y., Hamza A. & Ma E. Temperature-dependent strain rate sensitivity and activation volume of nanocrystalline Ni. Acta Mater. 54, 2715–2726 (2006).

[b6] Wei Y., Bower A. F. & Gao H. Enhanced strain-rate sensitivity in fcc nanocrystals due to grain-boundary diffusion and sliding. Acta Mater. 56, 1741–1752 (2008).

[b7] McFadden S. X., Mishra R. S., Valiev R. Z., Zhilyaev A. P. & Mukherjee A. K. Low-temperature superplasticity in nanostructured nickel and metal alloys. Nature 398, 684–686 (1999).

[b8] Rajagopalan J., Han J. H. & Saif M. T. A. Plastic Deformation Recovery in Freestanding Nanocrystalline Aluminum and Gold Thin Films. Science 315, 1831–1834 (2007). 17395826 10.1126/science.1137580

[b9] Wei X. & Kysar J. W. Residual plastic strain recovery driven by grain boundary diffusion in nanocrystalline thin films. Acta Mater. 59, 3937–3945 (2011).

[b10] Rajagopalan J., Han J. H. & Saif M. T. A. Bauschinger effect in unpassivated freestanding nanoscale metal films. Scr. Mater. 59, 734–737 (2008).

[b11] Espinosa H. D., Bernal R. A. & Filleter T. *In situ* TEM Electromechanical Testing of Nanowires and Nanotubes. Small 8, 3233–3252 (2012). 22903735 10.1002/smll.201200342

[b12] Shan Z. *et al.* Grain Boundary-Mediated Plasticity in Nanocrystalline Nickel. Science 305, 654–657 (2004). 15286368 10.1126/science.1098741

[b13] Shan Z. W., Mishra R. K., Syed Asif S. A., Warren O. L. & Minor A. M. Mechanical annealing and source-limited deformation in submicrometre-diameter Ni crystals. Nat. Mater. 7, 115–119 (2008). 18157134 10.1038/nmat2085

[b14] Oh S. H., Legros M., Kiener D. & Dehm G. *In situ* observation of dislocation nucleation and escape in a submicrometre aluminium single crystal. Nat. Mater. 8, 95–100 (2009). 19151703 10.1038/nmat2370

[b15] Legros M., Gianola D. S. & Motz C. Quantitative *In situ* mechanical testing in electron microscopes. MRS Bull. 35, 354–360 (2010).

[b16] Yu Q., Legros M. & Minor A. M. *In situ* TEM nanomechanics. MRS Bull. 40, 62–70 (2015).

[b17] Oh S. H. *et al.* Dislocation plasticity of Al film on polyimide investigated by cross-sectional *In situ* transmission electron microscopy straining. Scr. Mater. 65, 456–459 (2011).

[b18] Hugo R. C. *et al.* In-situ TEM tensile testing of DC magnetron sputtered and pulsed laser deposited Ni thin films. Acta Mater. 51, 1937–1943 (2003).

[b19] Kumar K. S., Suresh S., Chisholm M. F., Horton J. A. & Wang P. Deformation of electrodeposited nanocrystalline nickel. Acta Mater. 51, 387–405 (2003).

[b20] Hattar K., Han J., Saif M. T. A. & Robertson I. M. *In situ* Transmission Electron Microscopy Observations of Toughening Mechanisms in Ultra-fine Grained Columnar Aluminum Thin Films. J. Mater. Res. 20, 1869–1877 (2005).

[b21] Rajagopalan J., Rentenberger C., Peter Karnthaler H., Dehm G. & Saif M. T. A. *In situ* TEM study of microplasticity and Bauschinger effect in nanocrystalline metals. Acta Mater. 58, 4772–4782 (2010).

[b22] Mompiou F., Caillard D., Legros M. & Mughrabi H. *In situ* TEM observations of reverse dislocation motion upon unloading in tensile-deformed UFG aluminium. Acta Mater. 60, 3402–3414 (2012).

[b23] Wang Y. B., Li B. Q., Sui M. L. & Mao S. X. Deformation-induced grain rotation and growth in nanocrystalline Ni. Appl. Phys. Lett. 92, 011903 (2008).

[b24] Shan Z. *et al.* Large lattice strain in individual grains of deformed nanocrystalline Ni. Appl. Phys. Lett. 92, 091917 (2008).

[b25] Legros M., Gianola D. S. & Hemker K. J. *In situ* TEM observations of fast grain-boundary motion in stressed nanocrystalline aluminum films. Acta Mater. 56, 3380–3393 (2008).

[b26] Shan Z. *et al.* Dislocation Dynamics in Nanocrystalline Nickel. Phys. Rev. Lett. 98, (2007). 10.1103/PhysRevLett.98.095502 17359167

[b27] Wang L. *et al.* Grain rotation mediated by grain boundary dislocations in nanocrystalline platinum. Nat. Commun. 5, (2014). 10.1038/ncomms5402 PMC4109021 25030380

[b28] Mompiou F. *et al.* Inter- and intragranular plasticity mechanisms in ultrafine-grained Al thin films: An *In situ* TEM study. Acta Mater. 61, 205–216 (2013).

[b29] Kobler A., Kashiwar A., Hahn H. & Kübel C. Combination of *In situ* straining and ACOM TEM: A novel method for analysis of plastic deformation of nanocrystalline metals. Ultramicroscopy 128, 68–81 (2013). 23524380 10.1016/j.ultramic.2012.12.019

[b30] Rauch E. F. & Véron M. Automated crystal orientation and phase mapping in TEM. Mater. Charact. 98, 1–9 (2014).

[b31] Idrissi H. *et al.* Plasticity mechanisms in ultrafine grained freestanding aluminum thin films revealed by in-situ transmission electron microscopy nanomechanical testing. Appl. Phys. Lett. 104, 101903 (2014).

[b32] Egerton R. F., Li P. & Malac M. Radiation damage in the TEM and SEM. Micron 35, 399–409 (2004). 15120123 10.1016/j.micron.2004.02.003

[b33] Zheng K. *et al.* Electron-beam-assisted superplastic shaping of nanoscale amorphous silica. Nat. Commun. 1, 1–8 (2010). 20975693 10.1038/ncomms1021 PMC3047011

[b34] Han J. H., Rajagopalan J. & Saif M. T. A. MEMS-based testing stage to study electrical and mechanical properties of nanocrystalline metal films. Proc. SPIE, 64640C–64640C–8 (2007).

[b35] Dai S. *et al.* Electron-Beam-Induced Elastic–Plastic Transition in Si Nanowires. Nano Lett. 12, 2379–2385 (2012). 22494107 10.1021/nl3003528

[b36] Mingler B. & Karnthaler H. P. Radiation damage during HRTEM studies in pure Al and Al alloys. Int. J. Mater. Res. 97, 1041–1045 (2006).

[b37] Egerton R. F., McLeod R., Wang F. & Malac M. Basic questions related to electron-induced sputtering in the TEM. Ultramicroscopy 110, 991–997 (2010).

[b38] Bross H. & Haberlen O. The scattering of electrons by edge dislocations in Al. J. Phys. Condens. Matter 5, 7687 (1993).

[b39] Sprecher A. F., Mannan S. L. & Conrad H. Overview no. 49: On the mechanisms for the electroplastic effect in metals. Acta Metall. 34, 1145–1162 (1986).

[b40] Wang Z. Elastic and Inelastic Scattering in Electron Diffraction and Imaging. (Springer Science & Business Media, 2013).

[b41] Egerton R. F. Control of radiation damage in the TEM. Ultramicroscopy 127, 100–108 (2013). 22910614 10.1016/j.ultramic.2012.07.006

[b42] Williams D. B. & Carter C. B. Transmission Electron Microscopy: A Textbook for Materials Science. (Springer Science & Business Media, 2009).

[b43] Dong H., Wen B. & Melnik R. Relative importance of grain boundaries and size effects in thermal conductivity of nanocrystalline materials. Sci. Rep. 4, (2014). 10.1038/srep07037 PMC4229661 25391882

