# Supplementary material for: Electron Beam Induced Artifacts During in situ TEM Deformation of Nanostructured Metals
Source: Sci Rep. 2015 Nov 10;5:16345. doi: 10.1038/srep16345 (PMC4639785; doi:10.1038/srep16345)
Supplement: Supplementary Figure [file srep16345-s1.doc]

**Electron Beam Induced Artifacts During in situ TEM Deformation of Nanostructured Metals**

Rohit Sarkar^1^, Christian Rentenberger^2^, Jagannathan Rajagopalan^1^

1. School for Engineering of Matter Transport and Energy, Arizona State University, Tempe 85287, USA.

2. Physics of Nanostructured Materials, Faculty of Physics, University of Vienna, Boltzmanngasse 5, 1090 Vienna, Austria.

**Supplementary Figure 1:** Schematic of the deformation of thin films in our experiments. When uniaxial stress is applied, the film has a tendency to decrease its thickness rather than width because of constraints from sample geometry. The free surfaces along the y-direction (width) are farther apart, which constrain the film from contracting laterally in the x-y plane. This leads to a plane stress-like condition in the x-y plane, with both σ_x_ and σ_y_ positive, and causes more reduction in thickness.
