# Supplementary material for: Electron Beam Induced Artifacts During in situ TEM Deformation of Nanostructured Metals
Source: Sci Rep. 2015 Nov 10;5:16345. doi: 10.1038/srep16345 (PMC4639785; doi:10.1038/srep16345)
Supplement: Supplementary Video Captions [file srep16345-s2.doc]

**Supplementary Video Legends**

**videoS1:** The video shows dislocation activity in the Al-225 sample at 0.41% strain in the 3^rd^ cycle (green arrow in Fig 3a).

**videoS2:** The video shows dislocation activity in the Al-225 sample during unloading in the 6^th^ cycle (black arrow in Fig 3a).

**videoS3:** The video shows increased dislocation activity in the Al-225 film when the beam is shifted to a new location at the end of the 2^nd^ cycle (violet cross in Fig 3b) of the stress relaxation experiments. The first 22 seconds of the video correspond to the initial location. The beam is shifted to the new location at 30 seconds.

**videoS4:** The video shows increased dislocation activity in the Au film when the beam is shifted to a new location during the 3^rd^ cycle (green arrow in Fig 3d). A displacement pulse is applied around the 10 second mark. The first 50 seconds of the video correspond to the initial location. The beam is shifted to the new location at 55 seconds.
